# Impact of machine learning–driven analysis of blood transcriptomes in multiple sclerosis

**DOI:** 10.4103/NRR.NRR-D-25-00940

**Published:** 2025-11-25

**Authors:** Alessandro Digilio, Cinthia Farina

**Affiliations:** Institute of Experimental Neurology and Division of Neuroscience, IRCCS San Raffaele Scientific Institute, Milan, Italy

Multiple sclerosis (MS) is a chronic disorder of the central nervous system characterized by multifocal lesions where inflammation, demyelination, and neurodegeneration occur (Jakimovski et al., 2024). MS diagnosis primarily relies on the demonstration of dissemination in time and space of the lesions based on clinical, magnetic resonance imaging (MRI), and cerebrospinal fluid assessments (Jakimovski et al., 2024). The disease can follow distinct clinical trajectories broadly described as relapsing-remitting MS (RR MS), the most common form characterized by acute episodes of neurological worsening followed by partial or complete recovery, and primary progressive MS (PP MS), where neurological disability accumulates steadily from onset (Jakimovski et al., 2024). After several years of disease, RR MS patients may also develop a progressive course (thus called secondary progressive MS, SP MS) (Jakimovski et al., 2024). It is well known that animal models resembling MS are mostly immune-mediated, that MS is accompanied by alterations in the immune system, and that approved drugs are immunomodulatory or immunosuppressive (Jakimovski et al., 2024). These observations underline the key role of peripheral immunity in the pathogenesis and maintenance of a neurological disorder and have prompted attention toward peripheral biomarkers to capture early systemic signals of disease. In this evolving landscape, blood transcriptomics has emerged as a valuable and minimally invasive tool to explore immune changes in MS systematically. While early studies identified blood-based transcriptomic signatures at distinct MS forms by classical differential gene expression analyses (Srinivasan et al., 2017a, b), the development of machine learning (ML) algorithms to model human disorders has since revolutionized our approach to biomarker definition. Still, while the application of ML to clinical and MRI data has shown great limits in predicting MS diagnosis and evolution (Bonacchi et al., 2022), it has provided excellent results when trained with blood transcriptomic data (Acquaviva et al., 2020; Omrani et al., 2024).

**Figure 1:**
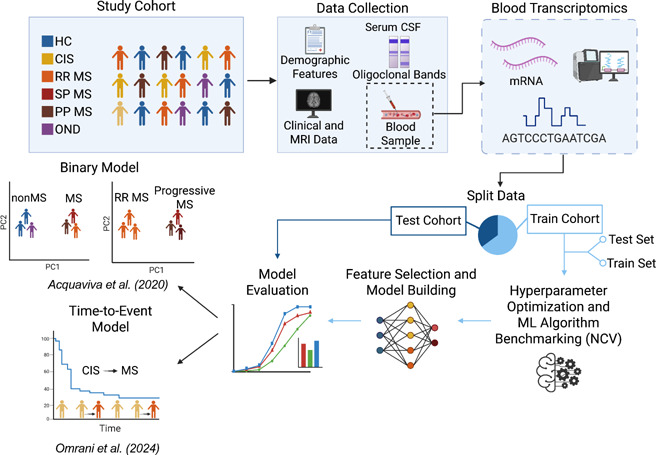
Schematic workflow of machine learning (ML)-based analysis of blood transcriptomes for multiple sclerosis (MS) modeling. Cohorts included healthy controls (HC), patients with a clinically isolated syndrome (CIS) or relapsing-remitting MS (RR MS) (adapted from Acquaviva et al., 2020; Omrani et al., 2024), and eventually secondary progressive MS (SP MS), primary progressive MS (PP MS), or other neurological disorders (OND) (adapted from Acquaviva et al., 2020). Demographic, clinical, magnetic resonance imaging (MRI), and paraclinical (oligoclonal bands in cerebrospinal fluid [CSF] *versus* serum) information was collected, and blood was drawn to generate blood/PBMC transcriptomes. Datasets were split into training and independent test sets. The training sets underwent nested cross-validation (NCV) for algorithm benchmarking, hyperparameter optimization, feature selection, and model generation. Models were then tested on the independent test sets. Binary models distinguished MS (RR MS, PP MS, and SP MS together) from non-MS (HC and OND together) and RR MS from progressive MS (adapted from Acquaviva et al., 2020), while time-to-event models incorporated CIS conversion time to generate individualized risk predictions for MS diagnosis (adapted from Omrani et al., 2024). Created with BioRender.com.

The initial efforts in this field focused on identifying molecular alterations in immune cells associated with different MS stages, applying differential gene expression approaches, to gain information on the underlying biology of MS and defining central regulators of dysimmunity and neuroinflammation (Srinivasan et al., 2017a, b; Colombo et al., 2023). In these studies, peripheral blood mononuclear cells (PBMC) were commonly used as an informative source of biological material, as they contain the main cell types implicated in MS pathogenesis (Jakimovski et al., 2024). Thus, expression analysis of MS risk or interferon-regulated genes across MS stages resulting from the comparison with signals from the healthy population uncovered that some parts of the dysregulated PBMC transcriptome were shared among MS forms (Srinivasan et al., 2017b), while others were specific for distinct clinical phenotypes. Starting from this observation, we hypothesized that transcriptional signals in PBMC could be exploited to discriminate among disease forms. To address this, we developed a complex statistical pipeline embedding machine learning algorithms to detect hidden patterns and non-linear relationships in complex high-dimensional data. A crucial requirement for reproducible ML modeling is the availability of a large number of samples and cases, which in our first study consisted of the whole PBMC transcriptomes from more than 300 human subjects, including healthy controls and patients with distinct MS forms or other neurological disorders (Acquaviva et al., 2020). This allowed us to split the cohort data into a train set and an independent test set (**Figure 1**). Distinct ML algorithms were compared using a nested cross-validation approach on the train set, which was also used for optimization of hyperparameters for some of the algorithms (**Figure 1**). This step was followed by the application of the optimized algorithms to the whole train set to extract the minimal features (i.e., transcripts) providing the best model on the training set, which was then verified on the independent test set (Acquaviva et al., 2020). Among tested algorithms, ADAboost-FT, an ensemble algorithm based on decision trees optimized for unbalanced classes, emerged as the top performer, distinguishing MS patients (RR MS, PP MS, and SP MS altogether) from controls (including healthy subjects and patients with other neurological disorders) with an accuracy of 87.5%. This evidence confirms the existence of a common transcriptional trait across disease forms useful to discriminate MS from controls. Notably, the same ML pipeline was extremely efficient in selecting features and building a model discriminating RR MS from progressive forms with accuracies exceeding 90% (Acquaviva et al., 2020). The biological features selected for the models referred mostly to genes involved in processes previously associated with MS (Acquaviva et al., 2020) but would not have been selected through conventional candidate-gene approaches, underscoring the added value of data-driven feature discovery. Overall, in this first study, we demonstrated the power of ML models based on PBMC transcriptomes in describing MS and its clinical forms.

In the next step, we moved from “state” models to truly predictive models and directed the analysis to whole blood transcriptomes to reduce sample processing, but with the risk of diluting PBMC information (Omrani et al., 2024). The key question we addressed was whether blood transcriptomes contain useful features to predict MS diagnosis. The disease begins with a first clinical manifestation suggestive of MS, referred to as clinically isolated syndrome, which converts to definite MS in case of further clinical and/or neuroradiological episodes (Marrie et al., 2022). The likelihood of MS diagnosis varies depending on the diagnostic criteria applied. This probability increased with the transition from the 2010 to the 2017 McDonald criteria, which, however, have low specificity, as they were developed to maximize therapeutic care (Filippi et al., 2022). Deep whole blood RNA sequencing was performed for more than 200 subjects, including treatment-naïve RR MS patients, clinically isolated syndrome patients, and sex- and age-matched healthy controls. As a first control experiment, we assessed whether whole blood transcriptomes were still a useful source of information to predict MS state by application of our binary classification pipeline to RR MS and healthy control data. The selected model showed 97% accuracy in distinguishing RR MS from the healthy population, confirming and extending to whole blood the power of ML-based recognition of MS state (Omrani et al., 2024). However, the same pipeline was ineffective in identifying clinically isolated syndrome patients and stratifying them. Considering that conversion time does not follow a binary distribution but is rather a continuous linear variable, we developed a novel ML pipeline incorporating conversion time into the construction of the predictive model (time-to-event modeling, **Figure 1**). After algorithm benchmarking and feature selection, the best model generated by the Random Survival Forest algorithm was based on a set of 58 transcriptomic features, mostly composed of regulatory RNA, including microRNA and long non-coding RNA (Omrani et al., 2024). Interestingly, only part of the predictive transcripts was differentially expressed, demonstrating the strength of data-driven approaches in capturing hidden molecular signals and indicating that prediction tasks also require signals, which are not passing classical statistical thresholds but still may be crucial for recognition of difficult cases. Importantly, the time-to-event pipeline generated personalized time-dependent risk prediction curves reaching an accuracy of 74.3% on the independent test cohort (Omrani et al., 2024). Application of the same framework to demographic (sex and age) and paraclinical (MRI and oligoclonal bands) data available at the first clinical event was less powerful (70.5% accuracy), demonstrating that blood RNA provides the best prediction for the diagnosis of a neurological disorder such as MS.

The search for a reliable biomarker in MS has long been a central goal in both research and clinical settings, driven by the need for tools supporting diagnosis, prognosis, and therapeutic decisions. In recent years, attention has increasingly shifted toward serum biomarkers such as the neurofilament light chain, which, however, lacks disease specificity and presents with high interindividual variability (Thebault et al., 2022). The biological heterogeneity of MS, together with its variable clinical manifestations and trajectories, has made the identification of a single, universally reliable biomarker particularly challenging. To move beyond this reductionist view, still common in clinical setting, we propose to develop integrative models that can capture and combine different types of information. While central nervous system biomarkers, as detected by MRI, are ineffective in stratifying clinical phenotypes to suggest a unified set of diagnostic criteria for RR MS and PP MS (Brownlee et al., 2025), blood transcriptional biomarkers are excellent biological correlates for clinical definitions of disease courses, as shown in our studies. This result cannot be achieved by the commonly used measurement of serum neurofilament light (Thebault et al., 2022). Further, unbiased data-driven selection of blood transcripts may highlight features that are not obvious and may generate novel knowledge about MS immunopathogenesis. Conversely, several issues need to be taken into consideration when performing transcriptomics studies. Given the dynamic nature of the blood transcriptome, it is important that inclusion criteria ensure the enrolment of subjects who are not affected by inflammatory and/or autoimmune conditions (except for the pathological condition of interest) and that blood is collected from treatment-naïve patients or after prolonged wash-out from therapy, as MS treatments have profound effects on immune cell composition and phenotype (Jakimovski et al., 2024), thereby altering the primary immune dysregulation associated with the disease. Regarding blood sample collection and processing, we commonly use PAXgene Blood RNA tubes for whole blood transcriptomics to stabilize RNA immediately (Omrani et al., 2024) or perform PBMC isolation by 30 minutes from blood collection into tubes with anticoagulants to avoid storage artifacts (Acquaviva et al., 2020). Another consideration is that, when sample collection and data generation occur along years as often occurs in clinical settings, batch effects may arise, e.g., from sample processing with many distinct lots of reagents and set-up of sequencing platforms. To overcome this issue, we commonly repeat measurements of technical replicates, estimate eventual batches and correct them while retaining biological variation, e.g., by ComBat (Acquaviva et al., 2020).

Future challenges include the development of ML-based blood transcriptomics for MS prognosis, a key issue for accurate therapeutic choices that require the identification of patients at high or low risk of disease activity and progression. For this purpose, model refinement and optimization may also benefit from the combination with other big data, like those generated by conventional and non-conventional MRI or other omics approaches such as serum/cerebrospinal fluid proteomics. Ultimately, MS may serve as a prototypical model for developing blood-based analytical frameworks for other neurological disorders, including Parkinson’s disease, amyotrophic lateral sclerosis, and Alzheimer’s disease, where immune dysregulations have also been documented (Zhang et al., 2023).

*This work was supported by Italian Ministry for Health (RF-2011-02349698, RF-2018-12367731) (to CF).*
